# Mapping the structural connectivity between the periaqueductal gray and the cerebellum in humans

**DOI:** 10.1007/s00429-019-01893-x

**Published:** 2019-06-05

**Authors:** Alberto Cacciola, Salvatore Bertino, Gianpaolo Antonio Basile, Debora Di Mauro, Alessandro Calamuneri, Gaetana Chillemi, Antonio Duca, Daniele Bruschetta, Paolo Flace, Angelo Favaloro, Rocco Salvatore Calabrò, Giuseppe Anastasi, Demetrio Milardi

**Affiliations:** 1 Department of Biomedical, Dental Sciences and Morphological and Functional Images, University of Messina, Messina, Italy; 2 IRCCS Centro Neurolesi “Bonino Pulejo”, Messina, Italy; 3 School of Medicine, University of Bari ‘Aldo Moro’, Bari, Italy

**Keywords:** Connectome, CSD, Diffusion MRI, White matter, Tractography

## Abstract

The periaqueductal gray is a mesencephalic structure involved in modulation of responses to stressful stimuli. Structural connections between the periaqueductal gray and the cerebellum have been described in animals and in a few diffusion tensor imaging studies. Nevertheless, these periaqueductal gray–cerebellum connectivity patterns have yet to be fully investigated in humans. The objective of this study was to qualitatively and quantitatively characterize such pathways using high-resolution, multi-shell data of 100 healthy subjects from the open-access Human Connectome Project repository combined with constrained spherical deconvolution probabilistic tractography. Our analysis revealed robust connectivity density profiles between the periaqueductal gray and cerebellar nuclei, especially with the fastigial nucleus, followed by the interposed and dentate nuclei. High-connectivity densities have been observed between vermal (Vermis IX, Vermis VIIIa, Vermis VIIIb, Vermis VI, Vermis X) and hemispheric cerebellar regions (Lobule IX). Our in vivo study provides for the first time insights on the organization of periaqueductal gray–cerebellar pathways thus opening new perspectives on cognitive, visceral and motor responses to threatening stimuli in humans.

## Introduction

The periaqueductal gray (PAG) is an important neuronal station situated in the mesencephalon, surrounding the Sylvian aqueduct. According to the present knowledge, it probably works as a main control station for innate and acquired responses to stressful stimuli such as fear, anxiety and pain, by coordinating and integrating appropriate vegetative and behavioral responses (Carrive 1993; Fanselow et al. 1991; Tovote et al. 2016; Walker and Carrive 2003). The current opinion is that PAG is a downstream regulatory station involved in a circuit including the prefrontal cortex (PFC), amygdala and hippocampus, exerting a descending control on the spinal cord (Bandler et al. 2000; Sokolowski and Corbin 2012; Tovote et al. 2015; Furlong et al. 2016). Although some of the aspects of this complex response to stressors are relatively well known (McMullan and Lumb 2006), the way PAG coordinates some complex motor responses, such as freezing behavior (Roelofs et al. 2010; Roelofs 2017), is still subject of debate.

Anatomical connectivity between PAG with the cerebellum has been subject of interest in the past decades and has been investigated by means of tract-tracing techniques (Chan-Palay 1977; Dietrichs 1983).

Animal studies demonstrated that PAG and cerebellum are connected both directly and indirectly (Watson et al. 2016). Connections joining the cerebellar cortex and nuclei with PAG were first described in monkeys (Chan-Palay 1977). A few years later, a direct connection between PAG and some cerebellar sub-regions was demonstrated using fiber tracers in cats (Dietrichs 1983) and, more recently, in rabbits, between PAG and flocculus folio P (Nisimaru et al. 2013). In addition, neurophysiological findings of a strong coupling between PAG and cerebellar functions have also been provided in rats (Koutsikou et al. 2014).

According to these evidences, PAG may modulate cerebellar activity via three distinct, either direct or indirect, ways: (1) by controlling sensory afferent spino-cerebellar projections; (2) by modulating the output response of cerebellar nuclei; (3) by regulating spinal reflex circuits (Cerminara et al. 2009; Koutsikou et al. 2014, 2015, 2017).

In humans, the development of novel magnetic resonance imaging (MRI) sequences and signal modeling techniques has provided important contributions to the study of the functional neuroanatomy of PAG (Menant et al. 2016). In this regard, functional MRI (fMRI) studies demonstrated high statistical dependencies between PAG and cerebellum both in healthy (Kong et al. 2010; Coulombe et al. 2016; Faull and Pattinson 2017) and pathological conditions (Case et al. 2017).

On the other hand, diffusion-weighted imaging (DWI) and tractography represent powerful tools to trace structural connections non-invasively and in vivo (Cacciola et al. 2016, 2017a, c, d, 2018, 2019; Milardi et al. 2016a, b, 2017; Arrigo et al. 2018; Calamuneri et al. 2018), by estimating diffusion properties of magnetically labeled water molecules along myelinated axons (Basser et al. 1994; Henderson 2012). Several tractography studies explored the structural connectivity of PAG in humans (Sillery et al. 2005; Hadjipavlou et al. 2006; Owen et al. 2007, 2008; Ezra et al. 2015), though only a few characterized either direct or indirect pathways between the PAG and cerebellum (Sillery et al. 2005; Hadjipavlou et al. 2006; Owen et al. 2008).

In particular, Sillery and colleagues (2005) found direct connections between PAG and cerebellum using probabilistic tractography with 1.5 T MRI on seven healthy subjects. Similar connections were described by Owen and coworkers (2008) in two out of four patients with deep brain stimulation (DBS) electrodes implanted in PAG for treating chronic pain.

However, to the best of our knowledge, none of these studies precisely characterized the topographical distributions of connections between the PAG and the cerebellar subregions. Therefore, aim of the present study was the systematic investigation and characterization of the structural connections between PAG and both the cerebellar cortex and nuclei. We employed multi-shell, high-angular resolution diffusion MRI (HARDI) data of 100 healthy subjects from the WU-Minn Human Connectome Project (HCP) repository combined with constrained spherical deconvolution (CSD) signal modeling. Herein, we provide comprehensive qualitative and quantitative descriptions of the connectivity patterns between the PAG and cerebellum.

## Materials and methods

### Subjects and data acquisition

High-quality structural and diffusion MRI data from the HCP repository have been employed. We obtained data for 100 healthy subjects (males = 46, females = 54 age range 22–36 years). Data were acquired by the Washington University, University of Minnesota, and Oxford University (WU-Minn) HCP Consortium (Van Essen et al. 2013). All the HCP subjects were scanned using a Siemens 3T Skyra scanner previously modified with a Siemens SC72 gradient coil and stronger gradient power supply with maximum gradient amplitude (Gmax) of 100 mT/m (initially 70 mT/m and 84 mT/m in the pilot phase), with the aim of improving diffusion imaging (Van Essen et al. 2013). The structural scans included T1-weighted acquisitions with the following parameters: TE = 2.14 ms, TR = 2400 ms, voxel size = 0.7 mm. (Uǧurbil et al. 2013). Diffusion-weighted images were acquired using a single-shot 2D spin-echo multiband echo planar imaging (EPI) sequence and equally distributed over three shells (*b*-values of 1000 s/mm^2^, 2000 s/mm^2^, and 3000 s/mm^2^), with isotropic spatial resolution of 1.25 mm (Sotiropoulos et al. 2013).

Data employed in this study were downloaded in the minimally pre-processed form consisting of: normalization of b_0_ image intensity across runs, registration of b_0_ images to T1w acquisition and other corrections, such as those for EPI susceptibility, eddy-current-induced distortions, gradient nonlinearities and subject motion (Glasser et al. 2013).

### MRI post-processing

Both structural and diffusion images were post-processed to perform tractography. Briefly, structural images underwent brain extraction (Smith 2002) and cortical and subcortical segmentation (Patenaude et al. 2011; Zhang et al. 2001) using BET, FAST and FIRST tools in FSL (Smith et al. 2004). The obtained masks were visually inspected and, if needed, modified by a trained neuroanatomist. A five-tissue segmented image was then obtained and used to run multi-shell multi-tissue CSD (MSMT-CSD), an improvement of CSD signal modelling technique, in which fiber orientation distribution function (fODF) is estimated directly from deconvolution of diffusion-weighted signal with a reference single-fiber response function (Tournier et al. 2007, 2008). The MSMT-CSD modelling technique represents a variant designed to support multi-shell data and to overcome classical CSD limitations when it comes to estimate fODF in the presence of tissue-type heterogeneity (Jeurissen et al. 2014). Estimation of fODF and tractography were performed using the MRtrix software (http://www.mrtrix.org) (Tournier et al. 2012).

### Region of interest (ROI) segmentation

To obtain useful ROIs for tractography (see paragraph below), both automated and semi-automated segmentation methods were used. The steps followed to obtain ROIs are listed in the following pipeline.

1. The ROIs were warped from MNI space to subject native space for each of our 100 subjects as follows: FSL’s FLIRT tool was applied to obtain a liner registration, and then a nonlinear registration (FSL’s FNIRT tool) was obtained from the affine registration of the previous step. The non-linear transformation was used to warp ROIs from MNI space to subject space.
2. The ROI of the PAG was obtained from the Keuken and Forstmann’s 7T atlas that provides ROIs obtained from high-resolution MP2RAGE and FLASH scans warped in MNI space (Keuken and Forstmann 2015) available at https://www.nitrc.org/projects/atag/. Once the ROI was resliced in the MNI space, a probability threshold of 50% was set employing the FSL’s command *fslmaths*.
3. Cerebellar ROIs were obtained using SUIT Atlas (http://www.diedrichsenlab.org/imaging/suit.html), a free probabilistic atlas of the human cerebellum in a dedicated space (SUIT space) designed to improve the alignment of infratentorial structures in respect to conventional MNI space (Diedrichsen et al. 2009, 2011; Diedrichsen 2006). We ran the segmentation pipeline for each subject using SUIT toolbox on SPM12 (Ashburner and Friston 2011) and obtained a large cerebellar ROI including cerebellar cortex and nuclei, from which we extracted all the cerebellar lobular and nuclear ROIs (Fig. 1).

**Fig. 1:**
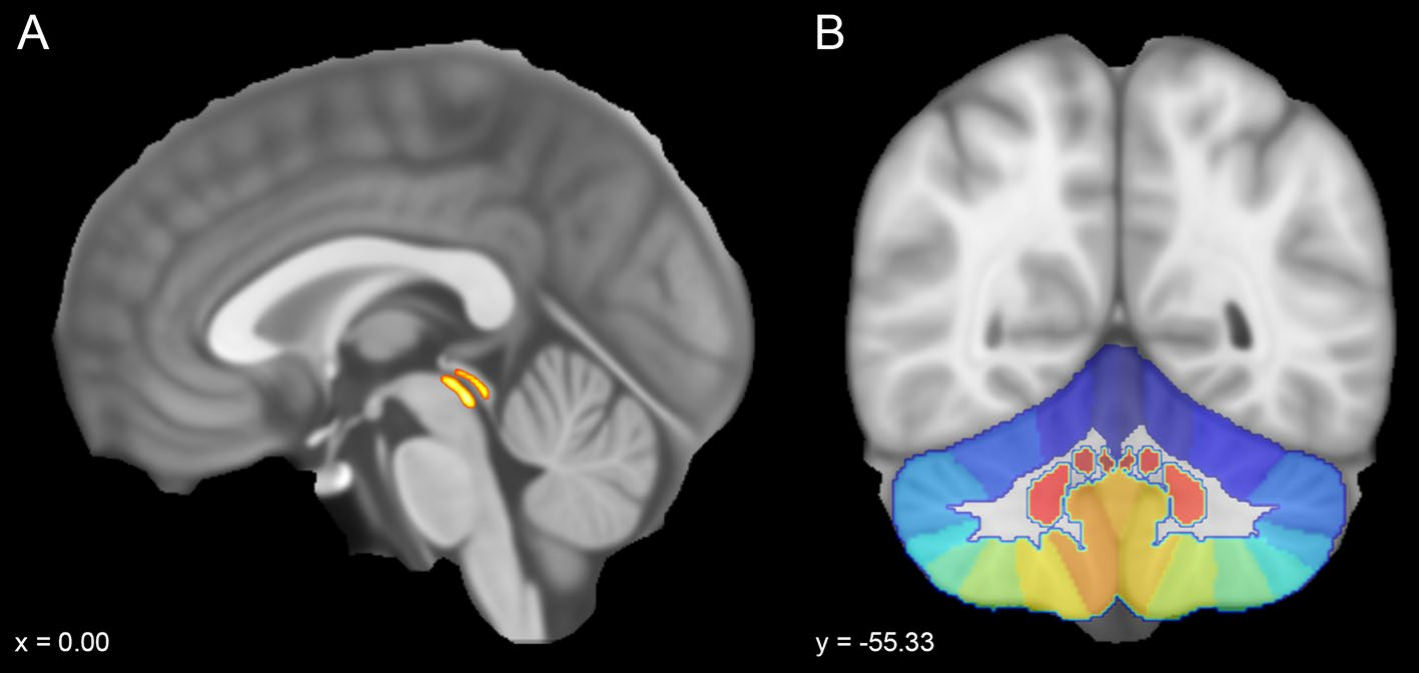
Sagittal and coronal sections of the MNI152 template (voxel size 0.5 mm). **a** ROI of the periaqueductal grey taken from the probabilistic atlas of the basal ganglia by Keuken and collegues was overlaid on the mid-sagittal plane. **b** Coronal view showing a colored scale of the vermal and hemispheric subregions provided by SUIT atlas
4. The cerebellum was parcellated in 34 regions of interest (six nuclei and 28 cerebellar cortex sub-regions including lobules and vermis) using SUIT (Diedrichsen et al. 2009). The whole PAG was considered as a median structure whilst the cerebellar cortex parcellation provided 12 subregions for each cerebellar hemisphere (Crus I, Crus II, Lobules I–IV, Lobule V, Lobule VI, Lobule VIIb, Lobule VIIIa, Lobule VIIIb, Lobule IX, Lobule X, dentate nucleus, interposed nucleus, fastigial nucleus), whereas the vermis was further subdivided in eight lobules (Vermis Crus I, Vermis Crus II, Vermis VI, Vermis VIIb, Vermis VIIIa, Vermis VIIIb, Vermis IX, Vermis X) (Fig. 1).
5. The cerebral grey matter (GM) previously obtained from FAST was also used as exclusion mask to filter out tracts that were not directed to cerebellar or brainstem grey matter, thus avoiding erroneous assignments (Verstynen et al. 2011, see below). The obtained grey matter mask was manually edited and then resliced into each subject’s native space using FLIRT tool on FSL.
6. Finally, since the high spatial proximity between PAG and the quadrigeminal plate (inferior and superior colliculi) could generate spurious tracts, e.g. from tectocerebellar pathways, we manually defined the quadrigeminal plate as region of avoidance (ROA). This ROA was outlined by a trained neuroanatomist on the MNI152 template and then warped it in subject’s native space using FLIRT tool on FSL.

All the aforementioned ROIs and ROAs were visually inspected and, if needed, manually modified for each subject by one of the authors.

### Tractography

Tractography was performed in the native space of each subject using the following pipeline: first, we reconstructed 1 million tracts using the PAG ROI as seed region, the large cerebellar ROI as inclusion mask (MRtrix’s -*include* option), the brain GM and quadrigeminal plate ROIs as exclusion masks (-*exclude* option) and IFod2 as fiber-tracking algorithm (Tournier et al. 2012).

In our study, spherical harmonic degree was fixed equal to six to obtain robustness to noise. During tractography, tracking was stopped in one of the following conditions: step size = 0.2 mm, maximum angle = 10°, minimal fODF amplitude = 0.15. This is a more conservative choice with respect to usual standards, since we preferred to underestimate fiber bundles to have more consistent reconstructions (Cacciola et al. 2017b; Descoteaux et al. 2009; Rizzo et al. 2018; Tournier et al. 2011). Once obtained 1 million streamlines between PAG and cerebellar ROI, tracts were “filtered out” using each single cerebellar lobular and nuclear ROI as inclusion mask and all the others as exclusion masks, and thus extracting single connections of interest between each cerebellar target region and PAG. It is worthy to note that we extensively used appropriate regions of avoidance (-*exclude* option) for an accurate extraction of streamlines of interest, and to avoid erroneous tract assignation (Verstynen et al. 2011).

### Connectivity analysis

Connectivity measures were obtained using in-house scripts built with MATLAB software package (http://www.mathworks.com), release 2015b. At first, the number of streamlines (NOS) connecting PAG to cerebellar regions was assessed. We defined connectivity density (*δ*) of each pathway of interest as the contribution of each target region, respectively, to the total NOS. With some limitations (Smith et al. 2013), such numbers are used as markers of connectivity density, both in healthy and pathological conditions (Behrens and Sporns 2012; Bijttebier et al. 2015; Guo et al. 2016; Zhang et al. 2017).

Since connectivity between seed region and regions of interest (ROI) is subjected to volume biases (Cheng et al. 2012), we extracted seed and target ROI volumes to scale the NOS by the mean volume of the two ROI involving each pathway thus computing a normalized connectivity density not affected from the volume bias (*δ*_NORM_). Hence, removing ROI volume contributions, we could estimate connectivity density profiles that are less sensitive to individual volumetric differences.

To summarize the distribution of the connectivity density for each reconstructed pathway, we computed the mean normalized density (*δ*_NORM_) and standard deviation (SD) from individual subject profiles.

Furthermore, for each connectivity density measure and for each pathway reconstructed, we assessed the inter-subject variability by means of coefficient of variation (COV), which was defined as the ratio of the SD to the *δ*_NORM_ estimated.

Finally, a lateralization index (LI) (Parker et al. 2005) was calculated for assessing lateralization in the investigated pathways as follows:

$$ {\text{LI}} = \frac{\text{Left} - \text{Right}}{\text{Left + Right}}.$$

Positive values of LI indicate left lateralization (LI > 0.1), whereas negative values indicate right lateralization (LI < 0.1). For each pathway, to assess statistically significant lateralization, permutation tests based on a *t*-statistic were performed using the connectivity profiles of each hemisphere gathered from each subject. 50.000 permutations were used to estimate the distribution of the null hypothesis, alpha level was set to 0.05, and the “tmax” method was adopted to correct the *p* values of each variable for multiple comparisons (Blair and Karkiski 1993).

## Results

To better summarize our results, we grouped cerebellar subregions following the structural and functional anatomical subdivision of the cerebellum described by Stoodley and Schamamann (2016). Lobules, vermal regions and deep cerebellar nuclei have been attributed to four compartments: (1) anterior cerebellum (Lobules I–IV, V), (2) posterior cerebellum (Lobules and Vermis VI, Crus I, Crus II, VIIb, VIIIa, VIIIb, IX), (3) flocculonodular lobe (Lobule and Vermis X), and (4) deep cerebellar nuclei (dentate nucleus, interposed nucleus and fastigial nucleus).

**Fig. 2:**
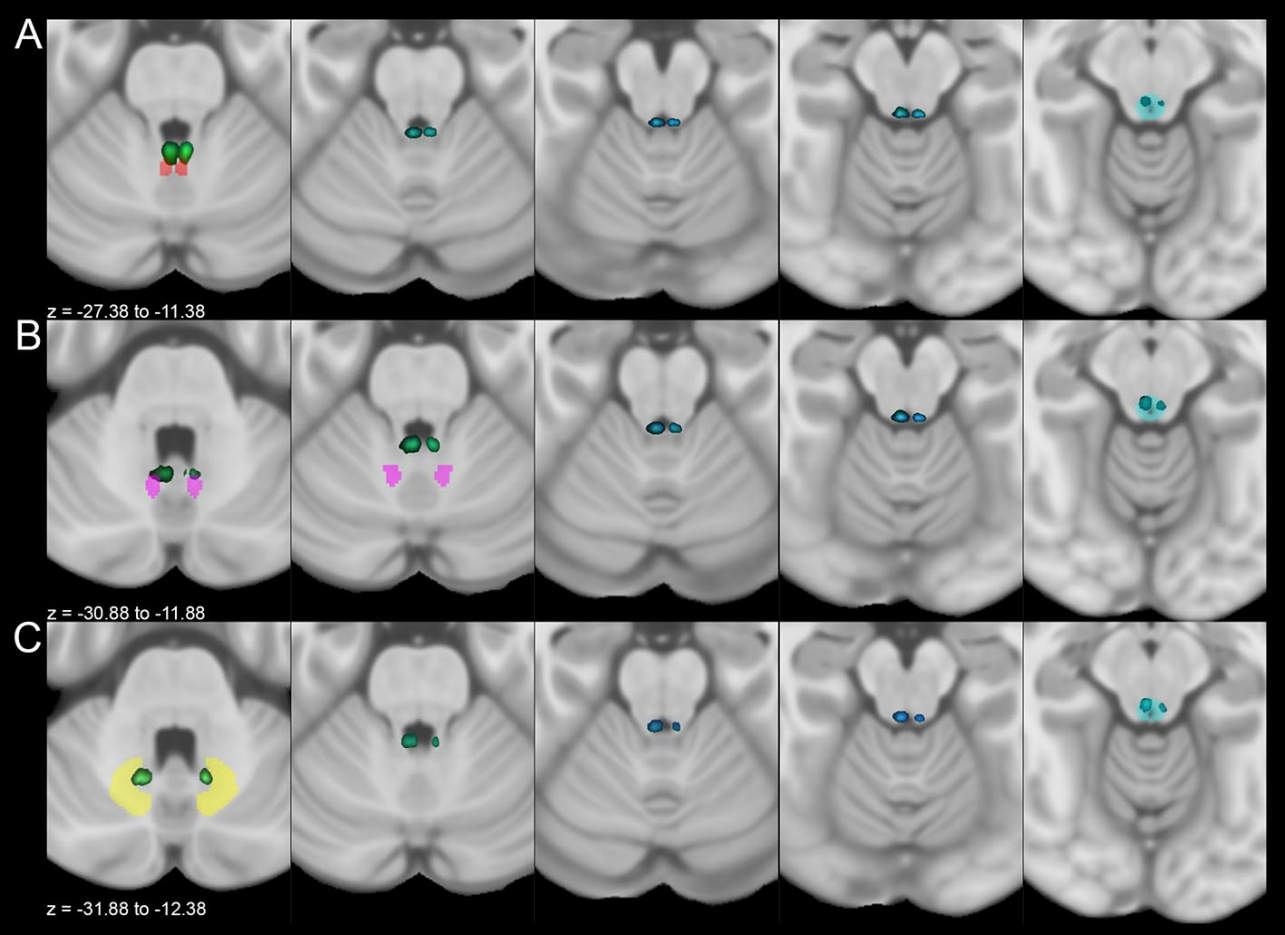
Average track density maps showing connections between cerebellar nuclei and periaqueductal grey mapped in directionally encoded color space (DEC) and superimposed to the MNI152 template. **a** Axial slices showing the course of tracts joining fastigial nucleus (red) and periaqueductal grey (cyan). The tracts leave the fastigial nucleus, pass through the superior cerebellar peduncle and reach the periaqueductal grey in the mesencephalon, sparing the superior colliculi. **b** Axial slices showing the course of tracts between interposed nucleus (pink) and periaqueductal grey (cyan). Tracts reach the mesencephalic periaqueductal grey via the superior cerebellar peduncle sparing the superior colliculi. **c** Axial slices representing the course of connections between dentate nucleus (yellow) and periaqueductal grey (cyan). The tracts leave the dentate nucleus, run through superior cerebellar peduncles and reach the periaqueductal grey avoiding superior colliculi

**Fig. 3:**
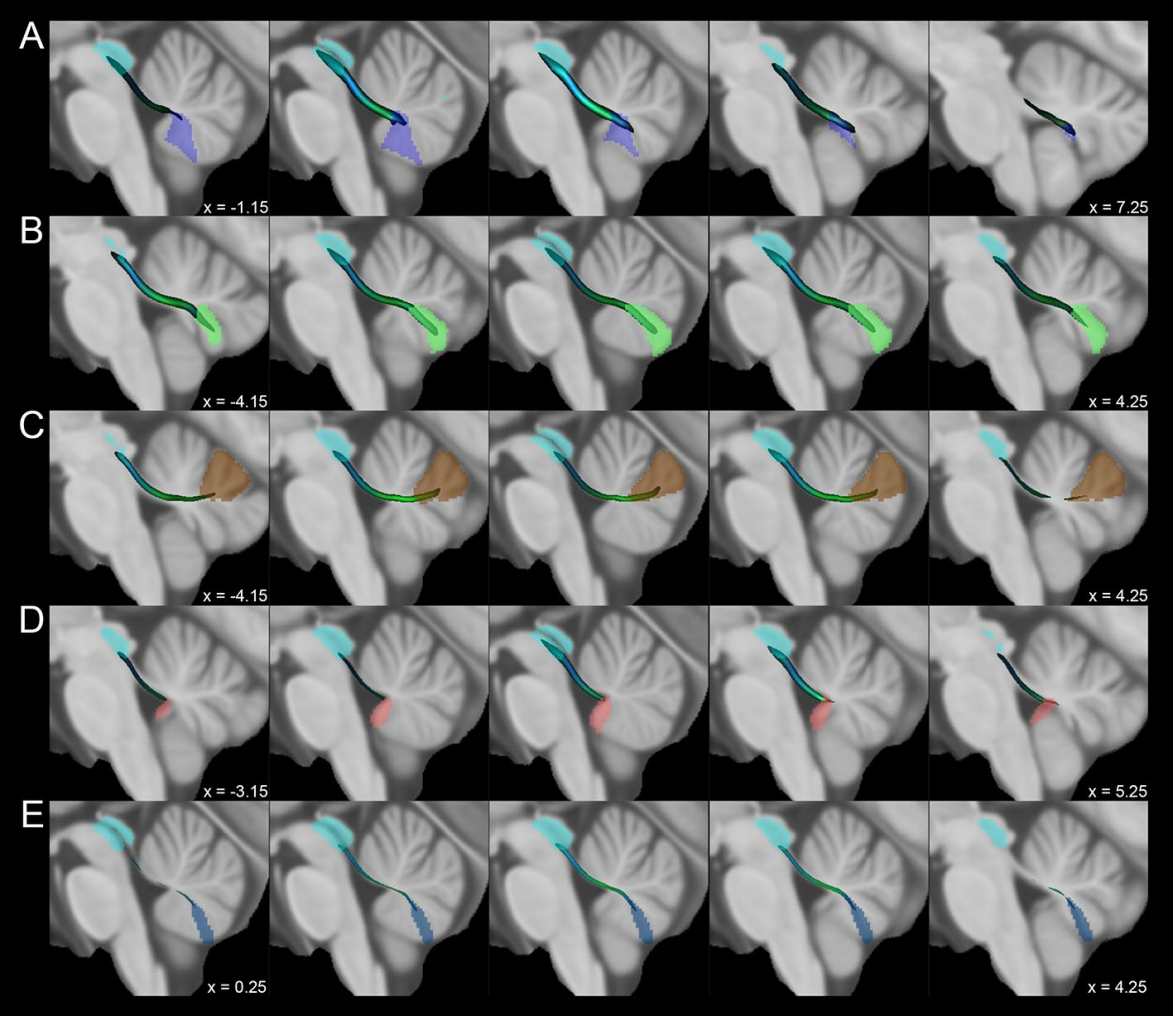
Average track density maps showing tracts connecting cerebellar vermal regions and periaqueductal grey mapped in directionally encoded color space (DEC) and superimposed to the MNI152 template. **a** Course of tracts connecting the Vermal lobule IX (violet) and the periaqueductal grey (cyan). **b** Connections between Vermal lobule VIIIa (green) and periaqueductal grey (cyan). **c** Tracts between Vermal lobule VI (brown) and periaqueductal grey (cyan). **d** Course of tracts joining Vermal lobule X (pink) to periaqueductal grey (cyan). **e** Connections between Vermal lobule VIIIb (blue) and periaqueductal grey (cyan)

**Fig. 4:**
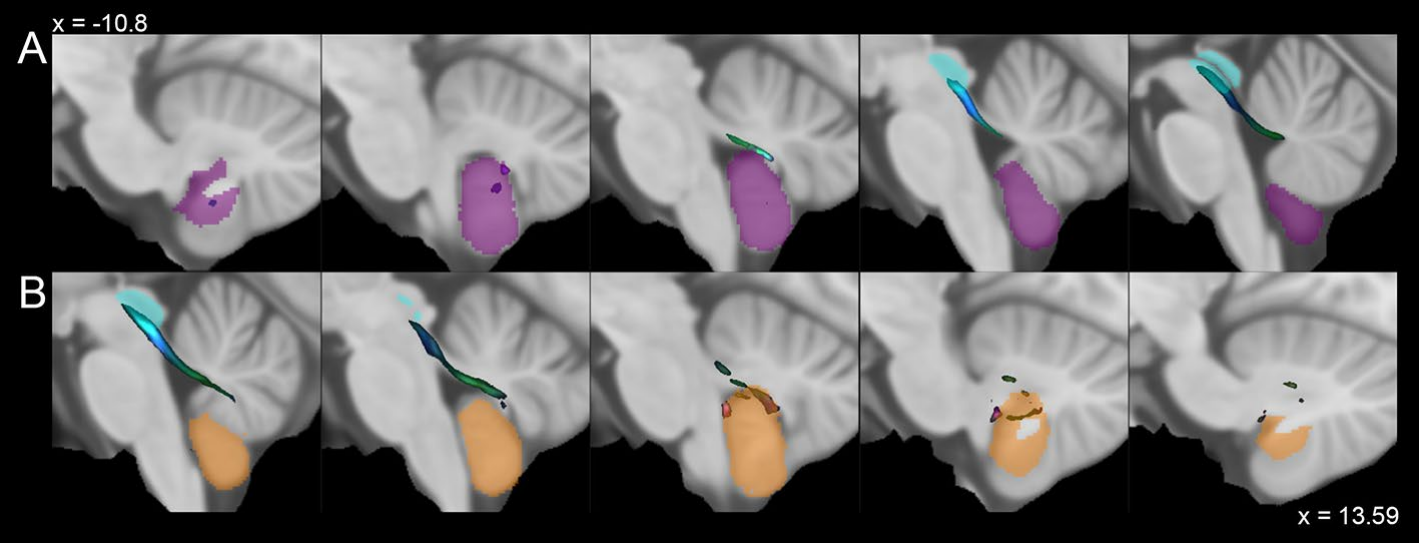
Average track density maps showing tracts connecting cerebellar hemispheric regions to periaqueductal grey mapped in directionally encoded color space (DEC) and superimposed to the MNI152 template. Sagittal views depicting tracts connecting **a** the left hemispheric Lobule IX (purple) and **b** the right hemispheric Lobule IX (orange) with the periaqueductal grey (cyan)

A first analysis focused on the morphological characterization of the fiber tracts connecting the PAG with the cerebellar structures. The streamlines arising from the PAG ran through the superior cerebellar peduncle and follow the arbor vitae of the cerebellum reaching nuclei, vermal regions and lobules. Connectivity patterns joining the PAG, respectively, with the deep cerebellar nuclei (Fig. 2), the entire vermis (Fig. 3) and lobules (Fig. 4) have been successfully reconstructed in all subjects.

A second analysis was finalized to assess quantitative structural connectivity by calculating the *δ*_NORM_ from connectivity data as described in the Connectivity analysis section. Consistent *δ*_NORM_ was found between PAG and all the cerebellar subregions across hemispheres, vermis and deep nuclei here considered. However, to provide more robust results, a probabilistic threshold (*δ*_NORM_ > 1%) has been applied to normalized tractograms to remove false-positive results (Rubinov and Sporns 2010). However, choosing a proper cut-off value remains quite empirical and still constitutes matter of debate (van Wijk et al. 2010).

Thus, only connections exceeding the probabilistic threshold *δ*_NORM_ > 1% of the whole PAG–cerebellum tracts were considered as true positives and will be object of discussion.

Robust connectivity patterns have been demonstrated between the PAG and cerebellar nuclei: the fastigial nucleus resulted to be the most connected (right: 17.2 ± 8.4%; left: 10.5 ± 5.4%), followed by the interposed nucleus (right: 10.7 ± 6.1%; left: 4.3 ± 3.6%) and the dentate nucleus (right: 4.5 ± 3.3%; left: 1.8 ± 1.6%) (Fig. 2, Table 1).

**Table 1:** Connectivity density (%) between the PAG and cerebellar structures

Normalized connectivity density			
Structure	Mean	SD	COV
Right fastigial nucleus	**17.24**	**8.41**	**0.49**
Vermis IX	**12.43**	**6.02**	**0.48**
Right interposed nucleus	**10.75**	**6.09**	**0.57**
Left fastigial nucleus	**10.47**	**5.44**	**0.52**
Vermis VIIIa	**9.90**	**5.38**	**0.54**
Right Lobule IX	**9.20**	**4.07**	**0.44**
Left Lobule IX	**5.81**	**2.97**	**0.51**
Vermis VI	**5.07**	**3.52**	**0.69**
Right dentate nucleus	**4.56**	**3.27**	**0.72**
Vermis X	**4.52**	**3.14**	**0.69**
Left interposed nucleus	**4.29**	**3.58**	**0.84**
Left dentate nucleus	**1.81**	**1.60**	**0.89**
Vermis VIIIb	**1.72**	**1.18**	**0.69**
Right Crus II	0.51	0.58	1.12
Right Lobule X	0.43	0.70	1.62
Left Lobule X	0.21	0.30	1.39
Left Crus II	0.18	0.20	1.10
Right Lobules I–IV	0.15	0.10	0.66
Right Lobule VIIIa	0.13	0.14	1.07
Left Lobule V	0.09	0.20	2.25
Right Lobule V	0.08	0.20	2.54
Right Lobule VIIb	0.07	0.09	1.31
Right Lobule VIIIb	0.07	0.13	1.89
Left Lobule VIIIb	0.07	0.08	1.20
Left Lobules I–IV	0.06	0.05	0.75
Left Lobule VIIIa	0.05	0.06	1.03
Right Crus I	0.04	0.06	1.63
Left Lobule VIIb	0.04	0.04	1.04
Vermis VIIb	0.02	0.03	1.44
Right Lobule VI	0.01	0.03	2.55
Left Crus I	0.01	0.02	1.25
Vermis Crus II	0.01	0.02	2.27
Left Lobule VI	0.01	0.01	1.96
Vermis Crus I	0.00	0.00	0.00

Connections exceeding the probabilistic threshold *δ*_NORM_ > 1% of the whole PAG–cerebellum tracts are reported in bold

*SD* standard deviation, *COV* coefficient of variation, *PAG* periaqueductal gray

The structures of the posterior compartment which showed consistent connectivity patterns with the PAG were the Vermis IX (12.4 ± 6.0%), Vermis VIIIa (9.9 ± 5.4%), Lobule IX (right: 9.2 ± 4.1%; left: 5.8% ± 3.0%), Vermis VI (5.1% ± 3.5%) and Vermis VIIIb (1.7 ± 1.2%), while Lobule VI, VIIIa, VIIIb, vermal and lobular Crus I, Crus II and VIIb showed an average *δ*_NORM_ < 1%. Connections between the PAG and flocculonodular lobe were also present with robust connections with the Vermis X (4.5 ± 3.1%) and weaker connectivity with Lobule X (< 1%) (Figs. 3 and 4).

The normalized connectivity density profiles are summarized in Table 1.

We also investigated the consistency of density percentages estimated from our subjects by looking at the COV. The most consistent results were obtained for the connections between the PAG and the right Lobule IX (COV = 0.44), the Vermis IX (COV = 0.48) and the right fastigial nucleus (COV = 0.49), whereas the highest variability between subjects was observed for the right dentate nucleus (COV = 0.72), the left interposed nucleus (COV = 0.84) and the left dentate nucleus (COV = 0.89).

It is worthy to note that highest COVs (one or above) often correspond to lowest values of mean *δ*_NORM_, mostly under the fixed threshold of 1%. This indicates that less connected regions are, at the same time, the most variable among subjects suggesting that the application of the aforementioned threshold could help to distinguish reliable from unreliable connections (Roberts et al. 2017).

A third analysis was carried out to assess the connectivity between PAG and each cerebellar compartment. The *δ*_NORM_ percentage revealed a prominent connectivity to nuclear region (49.10 ± 12.78%), followed by posterior (45.35 ± 10.24%), the flocculonodular (5.16 ± 3.23%) and anterior lobes (0.38 ± 0.30%).

Finally, the lateralization analysis, performed to assess differences between the connectivity profiles of PAG with the left and right cerebellar lobules and nuclei, revealed no significant side-to-side variations (*p* > 0.05).

## Discussion

**Fig. 5:**
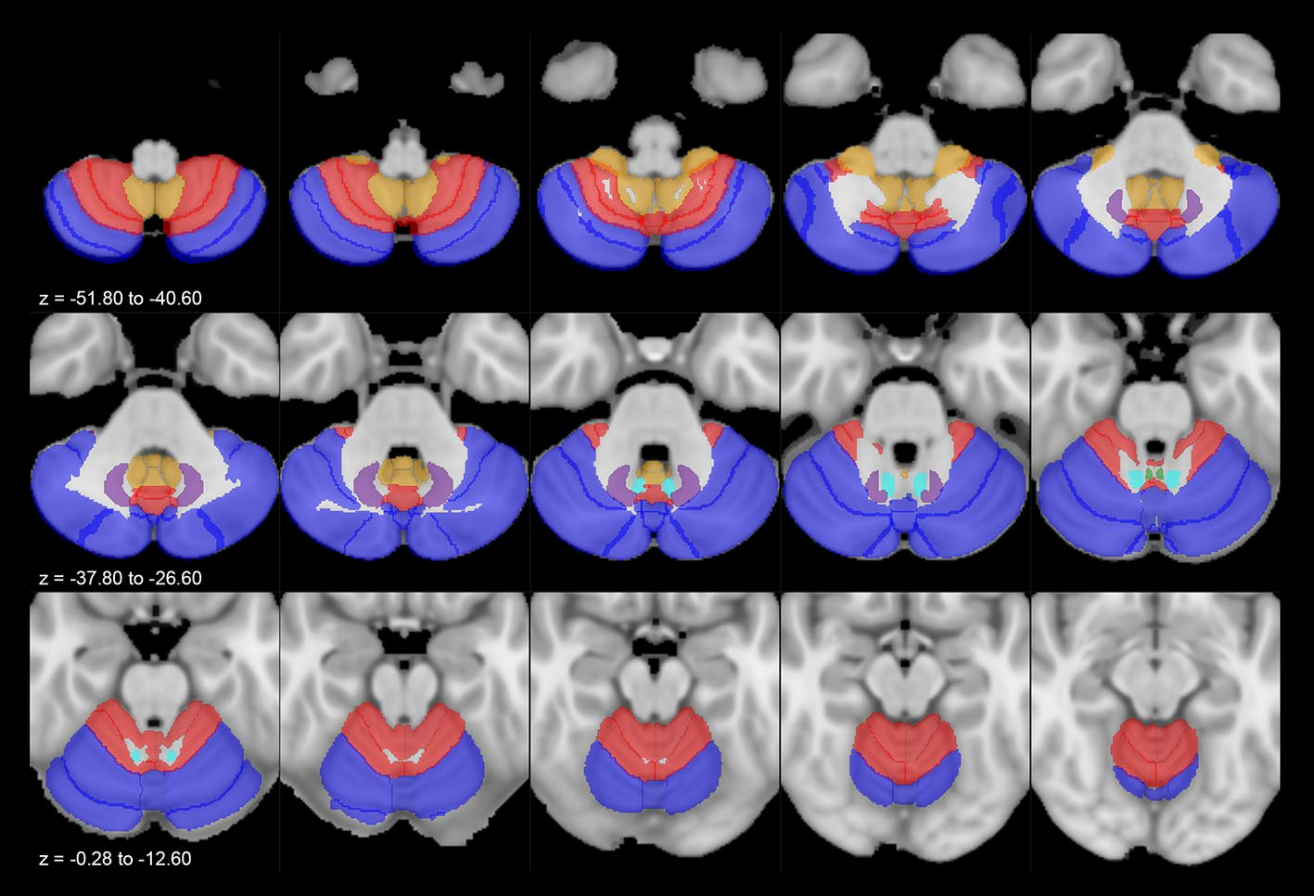
Multiple axial sections showing cerebellum lobules grouped as functional compartments: sensorimotor (red), cognitive/affective (blue), vestibular (yellow). Deep cerebellar nuclei have been labeled as follows: dentate in purple, interposed in cyan and fastigial in green

Our study aimed at providing new insights on the structural connectivity between the PAG and cerebellum performing MSMT-CSD tractography on high-resolution data from the WU-Minn HCP database. We observed that the PAG is highly connected with both the cerebellar cortex and deep cerebellar nuclei. To better summarize and discuss our findings, we will follow the structural and functional anatomical classification of the cerebellum proposed by Stoodley and Schmahmann (2009, 2010, 2016) (Fig. 5). The connectivity density profiles between PAG and cerebellar structures will be discussed following the structural classification, considering the existing literature regarding PAG–cerebellar connectivity (Table 2). The possible functional significance of the reconstructed connectivity patterns will be discussed in the last paragraph of the discussion.

**Table 2:** Evidences for the cerebellar-periaqueductal gray connectivity in animals and humans according to the existing literature

Authors (years)	Species	Method	Findings
Chan-Palay (1977)	Monkeys	Fiber tracing	Indirect PAG-olivo-cerebellar connections; direct fibers from dentate nucleus
Dietrichs (1983)	Cats	Fiber tracing	Direct connections from PAG to lobulus simplex, Crus I, Crus II, paramedian lobule and posterior lobule vermis
Teune et al. (2000)	Rats	Fiber tracing	Direct connections fibers from all cerebellar nuclei to PAG
Sillery et al. (2005)	Humans	Diffusion tensor imaging	Direct PAG–cerebellar connections
Owen et al. (2008)	Humans	Diffusion tensor imaging	Direct PAG–cerebellar connections based on pre-operative DTI for chronic pain
Cerminara et al. (2009)	Rats	Electrophysiology	Connections with paramedian lobule and copula pyramidis
Kong et al. (2010)	Humans	Resting-state functional MRI	High functional PAG–cerebellum connectivity (unspecified regions)
Moers-Hornikx et al. (2011)	Rats	Immunohistochemistry	Deactivation of deep cerebellar nuclei after PAG stimulation
Nisimaru et al. (2013)	Rabbits	Electrophysiology, immunohistochemistry	Hypothalamo-PAG-cerebellar neurons ending in flocculus folio P
Watson et al. (2013)	Rats	Electrophysiology	Indirect PAG-olivo-cerebellar connections
Koutsikou et al. (2014)	Rats	Electrophysiology	Connections with Vermis VIII and pyramis
Koutsikou et al. (2015)	Rats	Electrophysiology, immunohistochemistry	Connections with medial cerebellar nucleus
Coulombe et al. (2016)	Humans	Resting-state functional MRI	High PAG–cerebellum functional connectivity
Case et al. (2017)	Humans	Resting-state functional MRI	Higher functional PAG–cerebellum connectivity in sickle-cell disease patients vs controls
Faull and pattinson (2017)	Humans	Resting-state functional MRI	Functional connectivity with Lobules I–IV, V, VI, Crus I

### Deep cerebellar nuclei

Our connectivity analysis showed the highest strength for the connectivity patterns linking the PAG and the three deep cerebellar nuclei. While similar connections were reported in animals (Chan-Palay 1977; Koutsikou et al. 2015; Moers-Hornikx et al. 2011), to the best of our knowledge, our study represents the first evidence of the possible existence of the human analogues of these pathways in humans. The most connected nucleus to the PAG was the Fastigial nucleus, followed by the interposed and dentate nuclei (Fig. 2, Table 1). In rats, DBS of the dorsolateral column of PAG induces panic attacks and deactivates the deep cerebellar nuclei (Moers-Hornikx et al. 2011), whereas stimulation of the ventrolateral column of PAG induces Fos expression in fastigial (medial) nucleus (Koutsikou et al. 2015). It is worth to note that the majority of tracts between PAG and cerebellum reached the fastigial nucleus that, in addition to its well-known motor and oculomotor functions, is also involved in regulating complex vegetative responses such as cardiovascular tone, micturition, defecation, gastro-intestinal peristalsis and respiratory rate (Zhang et al. 2017) and is connected with limbic regions (Blatt et al. 2013). Moreover, the interposed nucleus, which retrieved high connectivity density values, also appears to play a role in fear conditioning (Sacchetti et al. 2002).

### Posterior cerebellum

Lower connectivity density profiles were observed between the PAG and the posterior lobe of the cerebellum. Interestingly, the highest values of connectivity for the regions belonging to posterior lobe were obtained for the uvula (Vermal lobule IX) and vermal pyramis (Vermal lobule VIIIa and VIIIb). The uvula is a well-known component of the vestibular cerebellum and receives afferents from vestibular receptors (Barmack 2003), exerting a well-recognized role in ocular movements (Voogd et al. 2012) and in postural adjustments via the regulation of the vestibulospinal system (McCall et al. 2017). On the other hand, the vermal pyramis is a component of the motor cerebellum: Vermal lobule VIII and the hemispheric Lobules VIII and IX are somatotopically organized constituting the posterior homunculus of the human cerebellum (Snider and Eldred 1951; Grodd et al. 2001). In rats, the electrical stimulation of the ventrolateral column of PAG leads to the activation of the vermal pyramis, eliciting fear-related freezing behavior, likely by influencing spinal descending neurons (Koutsikou et al. 2014). Finally, high-connectivity values were also found for Vermal lobule VI, a posterior vermis region that is part of the oculomotor vermis (Kheradmand and Zee 2011), but that also plays a role in the regulation of autonomic functions (Strata 2015) and in conditioned fear acquisition and retention (Sacchetti et al. 2004, 2007, 2009).

### Flocculonodular lobe

Finally, our probabilistic tractography study revealed connectivity patterns between the PAG and the flocculonodular lobe. Interestingly, the flocculonodular lobe reported the lowest connectivity values compared to the other lobes. It is worth to note that connections between PAG and flocculus folio P have been previously described as part of an hypothalamo-PAG-cerebellar pathway in rabbits (Nisimaru et al. 2013). As it is well known, together with uvula (Vermal lobule IX), lingula (Vermal lobules I–II) and the fastigial nucleus, the flocculonodular lobe is a key structure in the so-called paleocerebellum or vestibulocerebellum, involved in the regulation of ocular movements and posture by integrating vestibular afferences (Barmack 2003; McCall et al. 2017; Stoodley and Schmahmann 2016; Voogd et al. 2012; Zhang et al. 2017).

### Anatomo-functional considerations

Reactions to external threatening stimuli, such as fear and anxiety, require a complex regulation that involves the integration of cognitive, vegetative and motor responses. The PAG represents the main neural hub involved in top-down control of these responses to stressful or painful external stimuli.

One of such possible responses is the so-called freezing behavior, largely described in animals: the animal acquires a crouched posture (Blanchard and Blanchard 1969), increases its muscular tone, remains still and reduces vegetative parameters such as heart rate (Carrive 2000; Fanselow 1980, 1994; Kozlowska et al. 2015). As previously outlined, this response depends on PAG activation and is related to PAG–cerebellum interactions (Koutsikou et al. 2014). More recently, a similar response was described in humans (Hagenaars et al. 2014a, b; Roelofs 2017). In particular, reduced body sway and heart rate was observed in human individuals after the presentation of visual threatening stimuli such as emotionally significant pictures or films (Roelofs et al. 2010; Hagenaars et al. 2014a). However, the neuroanatomical substrates of this kind of response are still not clearly characterized in humans. Converging evidences from in vivo neuroimaging studies suggest that freezing behavior depends on prefrontal–amygdala–PAG functional connectivity (Mobbs et al. 2010; Hermans et al. 2013), but information is still lacking about the interface between the PAG and the motor system, that appear to be necessary to elicit such a complex and immediate postural response. Recently, PAG functional connectivity was evaluated during breathlessness, which is a multidimensional biopsychological condition that is associated with a strong affective component and feeling of fear and anxiety that could, in turn, lead to defensive responses such as freezing (Lansing et al. 2009; Herigstad et al. 2011; Hayen et al. 2013). A resting-state fMRI study found strong functional connectivity between the cerebellum and the ventrolateral column of PAG, in line with the present study and with previous animal findings. Interestingly, the same study performed task-related functional connectivity using psychophysiological interaction analysis in both breathlessness and breathlessness anticipation conditions, revealing that the strength of functional connectivity between the cerebellum and PAG is negatively correlated with the perceived intensity of breathlessness (Faull and Pattinson 2017). These data, thus, suggest an important role for the cerebellar–PAG interplay in coping responses to threatening stimuli, demonstrating how its “breakdown” could lead to a worse response to dangerous situations.

In this view, and in line with previous findings in animals, our results could be seen as a possible anatomical substrate for similar responses in humans. Indeed, we showed that the PAG is highly connected with the deep cerebellar nuclei, and especially with the fastigial and interposed nuclei, which play important roles in both motor and nonmotor vegetative functions such as autonomical regulation (Zhang et al. 2017) and fear conditioning (Sacchetti et al. 2002).

Moreover, robust connectivity density profiles were reported with motion- and posture-related cerebellar regions: the aforementioned deep cerebellar nuclei, vestibulocerebellar regions such as uvula and nodulus (Vermis IX–X), and vermal pyramis (VIIIa and VIIIb). Therefore, our results further reinforce the current idea of the involvement of posterior cerebellar vermis in fear conditioning, acquisition and retention (Sacchetti et al. 2002, 2004, 2007, 2009).

The structural connectivity patterns between the PAG and the aforementioned cerebellar regions could thus be hypothesized as part of a widespread network involved in processing fearful or noxious stimuli. However, these results should be interpreted with care, due to intrinsic limitations of the technique. Nevertheless, as a working hypothesis and trigger for further functional and structural investigations, we may suggest that such connections could represent the direct pathways through which the PAG and the cerebellum can cooperate for orchestrating complex responses to threatening stimuli.

## Limitations

This study is prone to limitations due to some intrinsic weaknesses of the technique which have been recently summarized in a recent review (Maier-Hein et al. 2017). Tractography deals with the axial symmetry of diffusion signal which prevents to distinguish afferent from efferent connections. At the same time, this technique is not able to the detect synapses, thus neglecting the recognition of monosynaptic pathways from polysynaptic ones. Consequently, inferences on the layer of the cerebellar cortex to which reconstructed fibers arrive are not allowed (Chung et al. 2011; Parker et al. 2013).

The diameter of axons is too tiny for MRI voxels; indeed, even at the highest definition, a single voxel contains thousands of axons, making the diffusion signal overestimated in respect to the scale of interest (Jbabdi and Johansen-Berg 2011).

Moreover, results reported in tractography studies are strongly influenced by different ways of modelling diffusion signal and depend on reconstruction parameters. An additional issue is represented by different fiber geometry (crossing, kinking, bending) resulting in very similar intra-voxel diffusion signal profiles, being instead different each other (Donahue et al. 2016b). Therefore, we employed a CSD-based signal modelling, together with restrictive reconstruction parameters, to overcome potential reconstruction biases that may be related to other modelling techniques (Dauguet et al. 2007; Descoteaux et al. 2009). Moreover, PAG–cerebellum connections not only have been described in animals via tract tracing techniques, but they have been reported by studies conducted on humans by diffusion tractography and fMRI.

The quantitative estimates of structural tractographic-based connectivity are still an open issue. As previously stated, an MRI voxel covers a multitude of axons; thus, the NOS is the most frequent measure employed to assess the strength of connections. However, although the NOS does not correspond to number of axons neglecting an anatomic-wise quantitative analysis (Jbabdi and Johansen-Berg 2011; Jones et al. 2013), a positive correlation between diffusion path probabilities and results of tract tracing studies has been recently demonstrated, thus validating the use of tractography-derived quantitative measures (Donahue et al. 2016a).

As it is well known, probabilistic tractography could lead to “false-positive” results and could thus overestimate quantitative connectivity measures (Jbabdi and Johansen-Berg 2011). Many authors in the field introduce a threshold, in the attempt to limit the effect of spurious tracking in probabilistic tractography (Rubinov and Sporns 2010; Drakesmith et al. 2015; Roberts et al. 2017). Therefore, tracts that do not contribute with a minimum percentage to the total tractogram are excluded. However, the choice of a proper threshold remains rather empirical and still constitutes subject of debate (van Wijk et al. 2010; Qi et al. 2015). Here, we employed a connectivity threshold > 1% to define tracts which consistently contributed to the total PAG–cerebellar streamlines. However, despite quite conservative and in line with previous studies, the threshold here employed remains arbitrary and should be acknowledged as an intrinsic limitation of the overall approach.

This is particularly important if we consider that several reconstructed tracts with *δ*_NORM_ < 1% showed a COV > 1, demonstrating a high variance in the connectivity density of these pathways among subjects.

Recently, Roberts et al. proposed the use of COV as an alternative connectivity measure for distinguishing less reliable from more reliable connections. This new approach would not underestimate long-range connections that usually show lower connectivity strength. In the present study, where only short range connection are taken into account, connectivity patterns with *δ*_NORM_ < 1% show the highest COVs suggesting that they may less likely have anatomical plausibility (Roberts et al. 2017).

Finally, despite employing high-definition T1-weighted scans at 0.75 × 0.75 × 0.75 mm resolution, we were not able to reach the necessary definition to distinguish the four longitudinal columns of the PAG and thus to characterize the columnar PAG connectivity.
